# A combination of genetic and biochemical analyses for the diagnosis of PI3K-AKT-mTOR pathway-associated megalencephaly

**DOI:** 10.1186/s12881-016-0363-6

**Published:** 2017-01-13

**Authors:** Yutaka Negishi, Fuyuki Miya, Ayako Hattori, Yoshikazu Johmura, Motoo Nakagawa, Naoki Ando, Ikumi Hori, Takao Togawa, Kohei Aoyama, Kei Ohashi, Shinobu Fukumura, Seiji Mizuno, Ayako Umemura, Yoko Kishimoto, Nobuhiko Okamoto, Mitsuhiro Kato, Tatsuhiko Tsunoda, Mami Yamasaki, Yonehiro Kanemura, Kenjiro Kosaki, Makoto Nakanishi, Shinji Saitoh

**Affiliations:** 1 Department of Pediatrics and Neonatology, Nagoya City University Graduate School of Medical Sciences, 1 Kawasumi, Mizuho-Cho, Mizuho-Ku, Nagoya, 467-8601 Japan; 2 Department of Medical Science Mathematics, Medical Research Institute, Tokyo Medical and Dental University, Tokyo, Japan; 3 Laboratory for Medical Science Mathematics, RIKEN Center for Integrative Medical Sciences, Yokohama, Japan; 4 Department of Cell Biology, Graduate School of Medical Sciences, Nagoya City University, Nagoya, Japan; 5 Department of Radiology, Nagoya City University Graduate School of Medical Sciences, Nagoya, Japan; 6 Department of Pediatrics, Sapporo Medical University School of Medicine, Sapporo, Japan; 7 Department of Pediatrics, Central Hospital, Aichi Human Service Center, Aichi, Japan; 8 Department of Pediatric Neurology, Central Hospital, Aichi Human Service Center, Aichi, Japan; 9 Department of Pediatrics, Shimada Ryoiku Center Hachiouji, Tokyo, Japan; 10 Department of Medical Genetics, Osaka Medical Center and Research Institute for Maternal and Child Health, Osaka, Japan; 11 Department of Pediatrics, Yamagata University Faculty of Medicine, Yamagata, Japan; 12 Department of Neurosurgery, Takatsuki General Hospital, Osaka, Japan; 13 Division of Regenerative Medicine, Institute for Clinical Research, Osaka National Hospital, National Hospital Organization, Osaka, Japan; 14 Department of Neurosurgery, Osaka National Hospital, National Hospital Organization, Osaka, Japan; 15 Center for Medical Genetics, Keio University School of Medicine, Tokyo, Japan; 16 Present address: Division of Cancer Cell Biology, Department of Cancer Biology, Instuite of Medical Science, The University of Tokyo, Tokyo, Japan; 17 Present address: Department of Pediatrics, Showa University School of Medicine, Tokyo, Japan

**Keywords:** *AKT3*, MCAP, MPPH, Multiplex targeted sequencing, Phosphorylated S6 ribosomal protein, *PIK3R2*, *PTEN*

## Abstract

**Background:**

Constitutive activation of the PI3K-AKT-mTOR pathway (mTOR pathway) underlies megalencephaly in many patients. Yet, prevalence of the involvement of the PI3K-AKT-mTOR pathway in patients with megalencephaly remains to be elucidated, and molecular diagnosis is challenging. Here, we have successfully established a combination of genetic and biochemical methods for diagnosis of mTOR pathway-associated megalencephaly, and have attempted to delineate the clinical characteristics of the disorder.

**Methods:**

Thirteen patients with an increased head circumference and neurological symptoms participated in the study. To evaluate the activation of the mTOR pathway, we performed western blot analysis to determine the expression levels of phosphorylated S6 ribosomal protein (phospho-S6 protein) in lymphoblastoid cell lines from 12 patients. Multiplex targeted sequencing analysis for 15 genes involved in the mTOR pathway was performed on 12 patients, and whole-exome sequencing was performed on one additional patient. Clinical features and MRI findings were also investigated.

**Results:**

We identified pathogenic mutations in six (*AKT3*, 1 patient; *PIK3R2*, 2 patients; *PTEN*, 3 patients) of the 13 patients. Increased expression of phospho-S6 protein was demonstrated in all five mutation-positive patients in whom western blotting was performed, as well as in three mutation-negative patients. Developmental delay, dysmorphic facial features were observed in almost all patients. Syndactyly/polydactyly and capillary malformations were not observed, even in patients with *AKT3* or *PIK3R2* mutations. There were no common phenotypes or MRI findings among these patients.

**Conclusions:**

A combination of genetic and biochemical methods successfully identified mTOR pathway involvement in nine of 13 (approximately 70%) patients with megalencephaly, indicating a major contribution of the pathway to the pathogenesis of megalencephaly. Our combined approach could be useful to identify patients who are suitable for future clinical trials using an mTOR inhibitor.

## Background

Megalencephaly is accompanied by hyperplasia of the brain parenchyma, and is defined by a head circumference greater than +2 SD from the mean of the general population. Various diseases involve development of the condition, including metabolic diseases, such as Alexander disease and Canavan disease, and syndromes, such as Sotos syndrome and Noonan syndrome [1].

In recent years, both megalencephaly-capillary malformation syndrome (MCAP, OMIM 602501) and megalencephaly-polymicrogyria-polydactyly-hydrocephalus syndrome (MPPH, OMIM 603387) have been shown to result from gain-of-function mutations in the PI3K-AKT-mTOR pathway (mTOR-pathway) [2]. MCAP and MPPH show very similar symptoms; the main symptoms are progressive megalencephaly, polymicrogyria, capillary malformations, syndactyly, and connective tissue dysplasia in the former [3–5], and progressive megalencephaly, polymicrogyria, and polydactyly in the latter [5–7]. Riviere et al. performed whole exome sequencing (WES) with a next-generation sequencer and identified germline mutations in *AKT3* and *PIK3R2* and a postzygotic mutation in *PIK3CA* in patients with MCAP, MPPH, and overlapping phenotypes of MCAP and MPPH [2]. Thereafter, Mirzaa et al. identified a germline mutation in *CCND2* in patients with MPPH [8]. Moreover, a loss-of-function mutation in *PTEN*, which suppresses the mTOR pathway, has been identified in autistic patients with macrocephaly [9, 10]. However, the prevalence of mTOR pathway involvement in patients with megalencephaly remains to be elucidated.

The mTOR pathway is involved in various functions including protein synthesis, lipid synthesis, autophagy, and energy metabolism, and is fundamental to essential biological functions. mTOR was originally discovered as a target protein for the immunosuppressant rapamycin [11, 12]. Rapamycin is used to treat tuberous sclerosis, a disease caused by mutations in either of the genes *TSC1* or *TSC2*, both of which are involved in the regulation of the mTOR pathway [13, 14]. Elucidation of the molecular mechanisms underlying megalencephaly is crucial to determine the value of investigating therapeutic agents, such as rapamycin, in the context of mTOR pathway-associated megalencephaly.

In this study, we conducted genetic and biochemical analyses in 13 patients with increased head circumference and neurological symptoms such as developmental delay and epilepsy, and investigated clinical features and imaging of mTOR pathway-associated megalencephaly.

## Methods

### Study subjects

We analyzed 13 patients with increased head circumference (>2 SD) and neurological symptoms such as developmental delay or epilepsy. These patients were included in this study, with a possibility of mTOR involvement after other diseases had been ruled out. All patients’ disease was sporadic. Clinical examination failed to make a specific diagnosis for each patient. Experimental protocols were approved by the Ethical Committee for the Study of Human Gene Analysis at Nagoya City University Graduate School of Medical Sciences (approval number 164). Written informed consent was obtained from all patients or their parents.

### Whole-exome sequencing

We performed WES in one parent-patient (Patient 1) trio because she had participated in another study of WES on brain malformation. To do this, genomic DNA was extracted from peripheral blood using the QIAamp DNA Blood Midi Kit according to the manufacturer’s instructions (Qiagen, Hilden, Germany). Genomic DNA was captured using the SureSelect XT Human All Exon V5 capture library (Agilent Technologies, Santa Clara, CA, USA), and sequenced using the Illumina HiSeq 2000 (Illumina, San Diego, CA, USA) with 100 bp paired-end reads. Exome data processing, variant calling and variant annotation were performed as described previously [15].

### Multiplex targeted sequencing

We performed multiplex targeted sequencing in 12 patients. Amplicon libraries of the target gene exons from 15 genes involved in the mTOR pathway were prepared with an Ion AmpliSeq Custom Panel (Thermo Fisher Scientific, Waltham, MA, USA). The number of exons, amplicons, and total targeted bases were 300, 412, and 43216 bases, respectively. This panel allowed theoretical coverage of 97.4% of the targeted sequences (Table 1). The library was prepared using the Ion AmpliSeq Library Kit 2.0 (Thermo Fisher Scientific), according to the manufacturer’s instructions. Emulsion PCR was performed using the Ion OneTouch system (Thermo Fisher Scientific) with the Ion OneTouch 200 Template Kit (Thermo Fisher Scientific), according to the manufacturer’s instructions. Multiplex targeted sequencing was performed with an Ion Torrent Personal Genome Machine (PGM) system using an Ion PGM 200 Sequencing Kit and an Ion 316 or 318 Chip (Thermo Fisher Scientific) according to the manufacturer’s instructions. Sequence data was analyzed using a CLC Genomic Workbench 7.0 (CLC bio, Aarhus, Denmark) and default settings.

**Table 1:** The panel of targeted genes involved in mTOR pathway

Gene	Chr	Number of exons	Number of amplicons	Total Bases	Overall coverage (%)
*PIK3CA*	3	20	34	3407	98.3
*PIK3CB*	3	22	30	3433	98.7
*PIK3CD*	1	22	37	3355	97.6
*PIK3R1*	5	18	23	2506	100
*PIK3R2*	19	15	19	2337	79.2
*PIK3R3*	1	10	14	1486	100
*PTEN*	10	9	12	1302	97.6
*PDPK1*	16	14	19	1811	95.3
*AKT1*	14	13	22	1573	97.5
*AKT2*	19	14	18	1687	98.9
*AKT3*	1	14	17	1624	98.8
*RHEB*	7	8	8	635	95.1
*MTOR*	1	57	69	8220	99.7
*TSC1*	9	22	29	3834	100
*TSC2*	6	42	61	6006	96.5
	Total	300	412	43216	97.4

*Chr* chromosome

Overall coverage means percent of coverage in target sequence

### Validation analysis by Sanger sequencing

We performed conventional Sanger sequencing to validate candidate mutations. We amplified the genomic regions by PCR (primer sequences are available on request), and directly sequenced using an ABI 310 Genetic Analyzer (Thermo Fisher Scientific), according to the manufacturer’s instructions.

### Mutation analysis of *CCND2*

We further sequenced the final exon of CCND2 in patients with no candidate mutation by Sanger sequencing.

### Lymphoblastoid cell lines

Epstein-Barr virus (EBV)-transformed lymphoblastoid cell lines (LCLs) were established from patients’ peripheral blood using a standard method. LCLs were cultured at 5% CO_2_ in RPMI medium (Sigma-Aldrich, Tokyo, Japan) with 10% FCS, L-glutamine and an antibiotic.

### Western blot analysis

Equal amounts of protein were boiled with SDS sample buffer (45 mmol/L Tris–HCl, pH 6.8, 10% glycerol, 1% SDS, 0.01% bromophenol blue, 50 mmol/L DTT). Proteins in the lysates were separated by SDS-PAGE and transferred to polyvinylidene difluoride (PVDF) membranes (Millipore, Billerica, MA, Japan). Membranes were blocked for 1 h with 5% dried skimmed milk in PBS with 0.1% Tween-20 (Sigma-Aldrich). Membranes were then incubated overnight with primary antibodies against phosphorylated S6 ribosomal protein (phospho-S6) (Ser240/244; diluted 1:1,000; Cell Signaling Technology, Danvers, MA, USA), and GAPDH (diluted 1:10,000; Cell Signaling Technology), followed by 1 h incubation with horseradish peroxidase–conjugated secondary antibody (GE Healthcare, Little Chalfont, UK). Bands were quantified using ImageJ, and the intensity of phospho-S6 was normalized with that of GAPDH. We obtained ratios to the normal control values. Five normal controls showed the ratio of 1.02 ± 0.17. Thus, we adopted 5xSD as cut-off and considered those of 2 or greater to be significantly increased and positive in this study.

## Results

WES of patient 1 generated 5.86 giga bases of nucleotide sequence. The average read depth of on-target regions was 69.3. This analysis identified a *de novo AKT3* heterozygous mutation [c.686A > G; p.(N229S)] (Table 2) that has been reported previously [2, 16–18], and is considered pathogenic.

**Table 2:** Genetic data, clinical features, and MRI findings of patients in this study

	Patient1	Patient2	Patient3	Patient4	Patient5	Patient6	Patient7	Patient8	Patient9	Patient10	Patient11	Patien12	Patient13
Age	3y0m	2y3m	6y8 m	4y2m	4y9m	4y6m	16y	4y8 m	7y4m	9y	2y10m	3y6m	2y0m
Sex	F	M	M	F	F	F	M	M	M	M	M	M	M
Elevated p-S6 protein (Relative density)	+ (3.4)	+ (3.0)	+ (3.8)	+ (3.6)	ND	+ (2.1)	+ (2.1)	+ (2.8)	+ (2.6)	- (0.8)	- (0.9)	- (1.1)	- (1.0)
Gene	*AKT3*	*PIK3R2*	*PIK3R2*	*PTEN*	*PTEN*	*PTEN*	-	-	-	-	-	-	-
Mutation	c.686A > G p.(N229S)	c.1117G > A p.(G373R)	c.1117G > A p.(G373R)	c.640C > T p.(Q214*)	c.740 T > C p.(L247S)	c.1006C > G p.(Y336*)	Unknown	Unknown	Unknown	Unknown	Unknown	Unknown	Unknown
Inheritance	de novo	de novo	de novo	Mother negative	de novo	de novo	-	-	-	-	-	-	-
Clinical features													
Gestational age	36w5d	36w6d	40w1d	37w4d	38w6d	39w5d	36w6d	36w5d	41w0d	38w1d	39w	38w6d	38w3d
Birth weight g (SD)^a^	2942 (+1.4)	3450 (+2.7)	3532 (+1.2)	2536 (−0.3)	2854 (+0)	3200 (+0.7)	2915 (+1.0)	3490 (+3.0)	2464 (−2.3)	3885 (+3.3)	3830 (+2.6)	4120 (+3.5)	3866 (+3.0)
Birth length cm (SD)^a^	47.5 (+0.3)	49.2 (+1.0)	52.5 (+1.4)	47 (−0.4)	50 (+0.7)	51 (+1.0)	48 (+0.4)	48.8 (+0.9)	47 (−1.6)	50.6 (+1.3)	NA	NA	48.4 (+0)
Birth OFC cm (SD)^a^	39 (+4.9)	36 (+2.5)	34 (+0.4)	33 (+0.1)	33 (−0.1)	34 (+0.5)	35 (+1.8)	37 (+3.3)	30 (−2.0)	41 (+6.4)	36.5 (+3.0)	37 (+3.0)	39 (+4.6)
Birth OFC percentile ^a^	>99	>99	67	53	44	70	96	>99	2	>99	>99	>99	>99
Last weight SD^b^	−1.9	0	−2.3	+0.4	−0.7	+0.8	−2.6	−0.2	−1.2	−0.2	+2.8	+2.2	+1.4
Last length SD^b^	−2.0	−0.2	−2.8	−0.4	−1.9	+0.2	−3.3	−1.3	−2.7	1.1	+0.7	−1.8	+0.2
Last OFC SD^c^	+6.3	+4.6	+3.0	+4.5	+4.3	+3.5	+4.9	+4.6	+2.0	+8.5	+5.4	+3.8	+4.5
Last OFC percentile ^c^	>99	>99	>99	>99	>99	>99	>99	>99	98	>99	>99	>99	>99
Overgrowth /Asymmetry	-	+	-	-	-	-	-	-	-	+	-	+	-
Vascular malformations	-	-	-	-	-	-	-	-	-	+	-	-	+
Syndactyly	-	-	-	-	-	-	-	-	-	-	-	-	-
Polydactyly	-	-	-	-	-	-	+	-	-	+	-	+	-
Connective tissue dysplasia	-	+	-	-	-	-	-	-	+	-	-	-	-
Dysmorphic features	+	+	+	+	+	+	+	+	+	+	+	+	+
DQ (assessed method) age	DD	DQ 42 (Denver) 10 m	DD	DQ 76 (KIDS) 2y5m	DQ 85 (KIDS) 4y6m	DQ 59 (K-test) 4y1m	DQ 12 (KIDS) 6y6m	DQ 71 (K-test) 6y8 m	DQ 35 (KIDS) 5y9m	DD	DQ 72 (K-test) 3y0m	DQ 46 (K-test) 1y7m	NA
Meaningful words	-	-	-	1y6m	8 m	2y6m	+	2y	-	+	2y	-	-
Walking alone	-	-	-	2y4m	2y2m	1y6m	+	1y11m	1y11m	4y6m	1y6m	2y3m	1y3m
Hypotonia	+	+	+	-	+	-	+	+	+	-	-	+	+
Seizure	+	-	+	-	-	-	+	-	-	-	-	-	-
MRI findings													
Ventriculomegaly	+	+	+	-	-	-	+	+	-	+	+	+	+
Polymicrogyria	+	+	+	-	-	-	+	-	-	+	-	+	-
Cerebellar tonsillar ectopia	-	-	-	-	-	-	-	+	-	+	-	+	-
White matter abnormalities	+	+	+	-	-	-	-	-	-	+	-	+	-

^a^SD and percentile were determined on the basis of the national data reported by the Ministry of Health, Welfare, and Labor in Japan in 2010

^b^SD was determined on the basis of the national data reported by the Ministry of Health, Welfare, and Labor in Japan in 2000

^c^SD and percentile were determined on the basis of CDC growth Charts for the United States in 2000

*DD* apparently developmentally delayed but not scored by a standardized method, *Denver* Denver Developmental Screening Test, *DQ* developmental quotient, *F* female, *KIDS* Kinder Infant Development Scale, *K-test* the revised version of Kyoto Scale of Psychological Development, *M* male, *NA* not available, *OFC* occipitofrontal circumference, *p-S6* phosphorylated S6 protein, *SD*, standard deviation

Accession number.: *AKT3*, NM_005465.4; *PIK3R2*, NM_005027.3; *PTEN*, NM_000314.5

Multiplex target next-generation sequencing was performed for another 12 patients. The median number of total sequenced bases per patient, of mapped reads, and of mean read length were 84.6 mega bases, 527 k reads, and 160 bases, respectively. The average read depth of the on-target regions was 1002-fold; 93.6% of the target regions had above 100-fold coverage. Using this multiplex targeted sequencing, a *de novo PIK3R2* heterozygous mutation [c.1117G > A; p.(G373R)] was identified in two patients, and *PTEN* heterozygous mutations [c.640C > T; p.(Q214*), c.740 T > C; p.(L247S), c.1006C > G; p.(Y336*)] were identified in three patients (Table 2). Five mutations in patients for whom both parents DNA were available for testing were confirmed to be *de novo.* Only a mother was available for testing one of the *PTEN* mutations [c.640C > T; p.(Q214*)], and was found to be negative (Table 2). The missense mutation in *PTEN* [c.740 T > C; p.(L247S)] was not reported previously. This *de novo* mutation was located in the C2 domain that is involved in binding to phospholipids in biological membranes [19], and was indicated to be “deleterious” and “possibly damaging” by in silico analysis with SIFT and PolyPhen-2, respectively [20, 21]. Thus, we considered it pathogenic according to the American College of Medical Genetics and Genomics interpretation guidelines [22]. Other mutations were identical to previously reported mutations, and were considered pathogenic [2, 23–25]. As shown in Table 3, the allelic frequency of the mutated allele detected by multiplex target next-generation sequencing was approximately 50%. Hence, all mutations were considered to be germline mutations.

**Table 3:** Mutant allele frequency in multiplex target next-generation sequencing

Subject	Gene	Mutation	Mutant allele	Total allele	%
Patient 1^a^	*AKT3*	p.(N229S)	43	78	55.1
Patient 2	*PIK3R2*	p.(G373R)	847	1828	46.3
Patient 3	*PIK3R2*	p.(G373R)	343	689	47.8
Patient 4	*PTEN*	p.(Q214*)	1497	2922	51.2
Patient 5	*PTEN*	p.(L247S)	238	500	47.6
Patient 6	*PTEN*	p.(Y336*)	96	214	44.9

^a^The mutation, which had previously been identified by WES, was reconfirmed by target next-generation sequencing

For the remaining seven patients in whom we did not detect any mutations by multiplex targeted sequencing, the last exon of *CCND2* was analyzed in addition, but no mutations were detected. *CCND2* is a recently described new MPPH gene [8], and the last exon corresponds to a mutational hotspot. *CCND2* was not included in the original targeted sequencing panel because it was reported after our panel was created. It is true that Sanger sequencing has limitations in the identification of somatic mutations. However, all previous *CCND2* mutations are considered to be heterozygous germline mutations, and thus at least major germline mutations in *CCND2* were excluded in our patients.

**Fig. 1:**
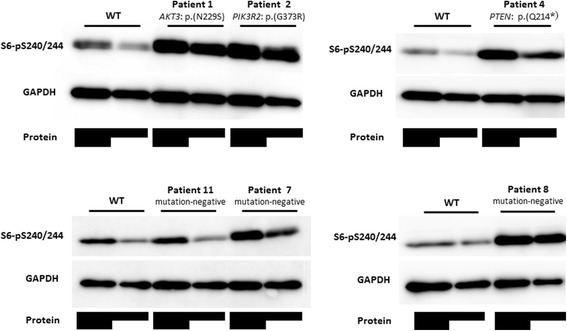
Representative western blot analysis of phospho-S6 protein levels in control and patient LCLs. Expression of phospho S6 (Ser 240/244) and GAPDH in variable amounts of protein extract (total protein indicated by shaded bars) are depicted in the upper and lower panel, respectively. After normalisation with GAPDH, there was increased expression of phospho-S6 in patients with mutations (Patient 1, 2, 4) and without mutations (Patient 7, 8), compared to wild type (WT) and a patient without mutations (Patient 11)

**Fig. 2:**
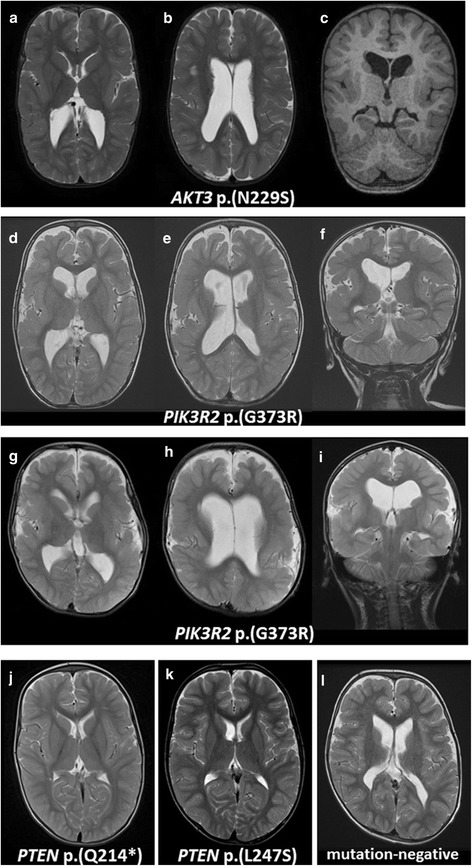
MRI findings in PI3K-AKT-mTOR pathway-associated megalencephaly. T2-weighted axial images (**a**, **b**, **d**, **e**, **g**, **h**, **j**-**l**), T1-weighted coronal image (**c**), T2-weighted images (**f**, **i**) of patients (**a**-**c**, Patient 1 with an *AKT3* mutation [p.(N229S)] at 2 years of age; **d**-**f**, Patient 2 with a *PIK3R2* mutation [p.(G373R)] at 2 years; **g**-**i**, Patient 3 with a *PIK3R2* mutation [p.(G373R)] at 6 years; **j**, Patient 4 with a *PTEN* mutation [p.(Q214*)] at 2 years; **k**, Patient 5 with a *PTEN* mutation [p.(L247S)] at 1 year and 9 months; **l**, Patient 8 without mutation at 4 years. In Patient 1–3, ventriculomegaly (**b**, **e**, **h**), bilateral polymicrogyria that appears to be most severe in the perisylvian regions but is also present in other regions (**a**, **c**, **d**, **f**, **g**, **i**), and an abnormally high intensity signal from white matter (**b**, **e**, **h**) were observed. The patient with *PTEN* mutations showed no abnormalities (**j**, **k**). Patient 8, without mutation, showed only mild ventriculomegaly (**l**)

Next, we analyzed the expression level of phospho-S6 protein in LCLs available from 12 patients by western blot analysis. Phospho-S6 protein lies downstream of the mTOR pathway, and is a marker of pathway activation [26, 27]. Of six patients for whom pathogenic mutations were identified, LCLs were established from five (LCL was not established for patient 5). All five patients showed an apparent increase in phospho-S6 protein expression. In addition, the expression level was also elevated in three of seven patients for whom no pathogenic mutations were identified (Fig. 1). Patients’ phenotypes are shown in Table 2. While all patients showed +2 SD or larger in head circumference, the head circumference at birth was not necessarily significantly large. Developmental delay, dysmorphic facial features including prominent forehead, long head, and ocular hypertelorism were observed in almost all patients. Syndactyly/polydactyly and capillary malformations, which are considered main symptoms of MCAP and MPPH, were not observed even in patients with *AKT3* or *PIK3R2* mutations. Regarding brain MRI findings, while ventriculomegaly, polymicrogyria and white matter abnormalities were observed in all patients with *AKT3* and *PIK3R2* mutations, no obvious abnormalities were found in patients with *PTEN* mutations (Fig. 2, Table 2). Phenotypes and MRI findings varied among the three mutation-negative patients who showed increased expression of phospho-S6 protein by western blot analysis. Moreover, no apparent phenotypic difference was noted between these patients and the patients with neither a pathogenic mutation, nor an increase in phospho-S6 expression.

## Discussion

In this study, we identified constitutive activation of the mTOR pathway in nine of 13 patients with megalencephaly of unknown etiology, indicating a significant role for the mTOR pathway in pathogenesis of genetic megalencephaly syndromes. We identified pathogenic mutations through multiplex targeted sequencing or WES in six patients. Of note is that all five mutation-positive patients in whom western blot analysis was performed showed abnormal activation of the mTOR pathway. Accordingly, we did not detect pathogenic mutations in four patients who did not show abnormal activation of the mTOR pathway. Thus, western blot analysis of phospho-S6 protein could be used as a biochemical marker to suggest megalencephaly associated with the constitutional activation of the mTOR pathway. It can also aid in confirming the results of molecular genetics analysis. Recently, Loconte et al. described three patients with *PIK3CA*-related overgrowth syndrome, including one patient with MCAP, and demonstrated the usefulness of a combination of genetic and biochemical methods [28]. They used skin fibroblasts for biochemical investigation, partially because some of their patients showed somatic mosaicism involving only limited parts of the limbs. Skin biopsy would be an important alternative method, particularly for patients with mosaicism, but it is not easily applied as an initial investigation. Our study demonstrated that blood could be used as a starting material to find possible involvement of the mTOR pathway.

In a report from Riviere et al., a *PIK3R2* mutation found at the same site [c.1117G > A; p.(G373R)] always presented clinical symptoms of MPPH. *PIK3CA* mutations were identified at multiple sites, and all but one case showed MCAP. *AKT3* mutations were found in patients with overlapping phenotypes or MPPH [2]. Thus, genotype and phenotype correlate considerably with each other. Nevertheless, syndactyly/polydactyly and capillary malformations, which are considered to be the main symptoms of both MCAP and MPPH, were not observed in patients with *AKT3* or *PIK3R2* mutations in our study. Mutations in *PTEN*, which suppresses the mTOR pathway, are also found in PTEN hamartoma syndrome, such as Cowden disease, Bannayan syndrome, and Proteus syndrome [19]. However, there is no evident genotype–phenotype association. The *PTEN* mutation [c.640C > T; p.(Q214*)] detected in our study has also been previously reported in Bannayan syndrome [23], but hamartomatous gastrointestinal polyposis or pigmented patches on the penis was not noted in our patient at the time of final evaluation. Another patient with a *PTEN* mutation also had no major anomaly other than megalencephaly and dysmorphic facial features. Neither patient had complications with tumors. While all patients showed a +2 SD or larger head circumference, the head circumference at birth was not necessarily large, indicating that megalencephaly in our patients was progressive. While developmental delays were observed in all patients, patients with *AKT3* or *PIK3R2* mutations had no meaningful words and had a more severe disease phenotype than those with *PTEN* mutations. Overall, no apparent differences in phenotype could be identified between patients with or without the involvement of the mTOR pathway and between patients with or without identified pathogenic mutations, suggesting genetic heterogeneity of the disorder.

Regarding head MRI findings, enlargement of the ventricle, polymicrogyria, and white matter abnormalities were observed in all patients with *AKT3* or *PIK3R2* mutations, and seemed indicative of mTOR pathway involvement. One mutation-negative patient, however, did exhibit these features, and thus these findings are not sufficiently specific to indicate all underlying pathogenesis. No abnormal MRI findings were observed in patients with *PTEN* mutations. The lack of a cortical anomaly has been reported for *PTEN* macrocephaly [10], and it appears to be an indicator of *PTEN* involvement.

In our study, target sequencing was performed primarily on 15 genes involved in the mTOR pathway. Therefore, the first conceivable limitation is the possibility of mutations in other genes that were not covered by our gene panel. In addition, our gene panel do not cover the entire exon region (the coverage ratio of *PIK3R2* in particular is low at 79.2%), and the Ion PGM method offers only limited indel detection. Nevertheless, our target sequencing is useful in early diagnosis in clinical settings due to the lower costs and shorter turn-around time than WES. Secondly, our strategy is limited when analyzing cases of mosaicism. Indeed, Riviere et al. performed targeted ultra-deep sequencing (coverage of more than 10,000 reads) of the *PIK3CA* mutation sites in mutation-negative affected individuals, as well as in known mutation carriers and control individuals [2]. This experiment detected additional low-level mosaic mutations missed by Sanger sequencing. Although a 100-fold or greater depth was obtained for about 93% of the target area with our target sequencing method, it is still possible that low-level mosaics were not detectable using this approach. Ultra-deep sequencing could be performed to identify such low-level mosaicism. Our biochemical analysis also used materials from peripheral blood, and thus it could not identify activation of the mTOR pathway in patients with mosaicism. Riviere et al. reported that mTOR pathway activation was also confirmed by means of western blot analysis using LCLs with low-level mosaics of a mutated allele frequency of 16% in the peripheral blood [2]. However, it is unknown whether similar results could be obtained with our protocol. It is also unknown whether similar results can be obtained in patients with lower allelic frequencies of the mutated allele. In rare cases, low-level mosaic mutations are detected only in the saliva, buccal mucosa, and LCLs. Therefore, affected tissues need to be used for molecular diagnosis of patients with mosaicism. The final limitation of this study is the small sample size. Further studies with larger sample sizes are warranted to perform WES or ultra-deep sequencing in patients who are positive with western blot analysis but negative with genetic analysis.

mTOR inhibitors are effective treatment for not only cancer, but for the brain and renal tumors of tuberous sclerosis [14]. They are also effective for epilepsy [13]. Moreover, their effects on intellectual disability and autism have also been demonstrated in experiments using model animals [29, 30]. Thus, the mTOR pathway associated-megalencephaly, a disease for which there is no curative treatment available, could also be a therapeutic target. Seizure related death was reported in a patient with mTOR pathway-associated megalencephaly [7], and thus development of a specific therapeutic intervention is crucial.

## Conclusions

A combination of genetic and biochemical methods was able to identify the involvement of the mTOR pathway in approximately 70% of patients with megalencephaly of an unknown etiology. Our combined approach could be useful to identify patients who are suitable for future clinical trials using an mTOR inhibitor.

## Abbreviations

LCL: Lymphoblastoid cell line
MCAP: Megalencephaly-capillary malformation syndrome
MPPH: Megalencephaly-polymicrogyria-polydactyly-hydrocephalus syndrome
MRI: Magnetic resonance imaging
mTOR: Mechanistic target of rapamycin
SD: Standard deviation
WES: Whole exome sequencing

## References

[1] Williams CA, Dagli A, Battaglia A (2008). Genetic disorders associated with macrocephaly. Am J Med Genet A.

[2] Riviere JB, Mirzaa GM, O’Roak BJ, Beddaoui M, Alcantara D, Conway RL, St-Onge J, Schwartzentruber JA, Gripp KW, Nikkel SM (2012). De novo germline and postzygotic mutations in AKT3, PIK3R2 and PIK3CA cause a spectrum of related megalencephaly syndromes. Nat Genet.

[3] Clayton-Smith J, Kerr B, Brunner H, Tranebjaerg L, Magee A, Hennekam RC, Mueller RF, Brueton L, Super M, Steen-Johnsen J (1997). Macrocephaly with cutis marmorata, haemangioma and syndactyly--a distinctive overgrowth syndrome. Clin Dysmorphol.

[4] Moore CA, Toriello HV, Abuelo DN, Bull MJ, Curry CJ, Hall BD, Higgins JV, Stevens CA, Twersky S, Weksberg R (1997). Macrocephaly-cutis marmorata telangiectatica congenita: a distinct disorder with developmental delay and connective tissue abnormalities. Am J Med Genet.

[5] Mirzaa GM, Conway RL, Gripp KW, Lerman-Sagie T, Siegel DH, deVries LS, Lev D, Kramer N, Hopkins E, Graham JM (2012). Megalencephaly-capillary malformation (MCAP) and megalencephaly-polydactyly-polymicrogyria-hydrocephalus (MPPH) syndromes: two closely related disorders of brain overgrowth and abnormal brain and body morphogenesis. Am J Med Genet A.

[6] Garavelli L, Leask K, Zanacca C, Pedori S, Albertini G, Della Giustina E, Croci GF, Magnani C, Banchini G, Clayton-Smith J (2005). MRI and neurological findings in macrocephaly-cutis marmorata telangiectatica congenita syndrome: report of ten cases and review of the literature. Genet Couns.

[7] Conway RL, Pressman BD, Dobyns WB, Danielpour M, Lee J, Sanchez-Lara PA, Butler MG, Zackai E, Campbell L, Saitta SC (2007). Neuroimaging findings in macrocephaly-capillary malformation: a longitudinal study of 17 patients. Am J Med Genet A.

[8] Mirzaa GM, Parry DA, Fry AE, Giamanco KA, Schwartzentruber J, Vanstone M, Logan CV, Roberts N, Johnson CA, Singh S (2014). De novo CCND2 mutations leading to stabilization of cyclin D2 cause megalencephaly-polymicrogyria-polydactyly-hydrocephalus syndrome. Nat Genet.

[9] Varga EA, Pastore M, Prior T, Herman GE, McBride KL (2009). The prevalence of PTEN mutations in a clinical pediatric cohort with autism spectrum disorders, developmental delay, and macrocephaly. Genet Med.

[10] McBride KL, Varga EA, Pastore MT, Prior TW, Manickam K, Atkin JF, Herman GE (2010). Confirmation study of PTEN mutations among individuals with autism or developmental delays/mental retardation and macrocephaly. Autism Res.

[11] Laplante M, Sabatini DM (2012). mTOR signaling in growth control and disease. Cell.

[12] Takei N, Nawa H (2014). mTOR signaling and its roles in normal and abnormal brain development. Front Mol Neurosci.

[13] Wong M (2010). Mammalian target of rapamycin (mTOR) inhibition as a potential antiepileptogenic therapy: from tuberous sclerosis to common acquired epilepsies. Epilepsia.

[14] Sampson JR (2009). Therapeutic targeting of mTOR in tuberous sclerosis. Biochem Soc Trans.

[15] Negishi Y, Miya F, Hattori A, Mizuno K, Hori I, Ando N, Okamoto N, Kato M, Tsunoda T, Yamasaki M (2015). Truncating mutation in NFIA causes brain malformation and urinary tract defects. Human Genome Variation.

[16] Nellist M, Schot R, Hoogeveen-Westerveld M, Neuteboom RF, van der Louw EJ, Lequin MH, Bindels-de Heus K, Sibbles BJ, de Coo R, Brooks A (2015). Germline activating AKT3 mutation associated with megalencephaly, polymicrogyria, epilepsy and hypoglycemia. Mol Genet Metab.

[17] Harada A, Miya F, Utsunomiya H, Kato M, Yamanaka T, Tsunoda T, Kosaki K, Kanemura Y, Yamasaki M (2015). Sudden death in a case of megalencephaly capillary malformation associated with a de novo mutation in AKT3. Childs Nerv Syst.

[18] Nakamura K, Kato M, Tohyama J, Shiohama T, Hayasaka K, Nishiyama K, Kodera H, Nakashima M, Tsurusaki Y, Miyake N (2014). AKT3 and PIK3R2 mutations in two patients with megalencephaly-related syndromes: MCAP and MPPH. Clin Genet.

[19] Eng C (2003). PTEN: one gene, many syndromes. Hum Mutat.

[20] Adzhubei IA, Schmidt S, Peshkin L, Ramensky VE, Gerasimova A, Bork P, Kondrashov AS, Sunyaev SR (2010). A method and server for predicting damaging missense mutations. Nat Methods.

[21] Kumar P, Henikoff S, Ng PC (2009). Predicting the effects of coding non-synonymous variants on protein function using the SIFT algorithm. Nat Protoc.

[22] Richards S, Aziz N, Bale S, Bick D, Das S, Gastier-Foster J, Grody WW, Hegde M, Lyon E, Spector E (2015). Standards and guidelines for the interpretation of sequence variants: a joint consensus recommendation of the American College of Medical Genetics and Genomics and the Association for Molecular Pathology. Genet Med.

[23] Longy M, Coulon V, Duboue B, David A, Larregue M, Eng C, Amati P, Kraimps JL, Bottani A, Lacombe D (1998). Mutations of PTEN in patients with bannayan-riley-ruvalcaba phenotype. J Med Genet.

[24] Tapper WJ, Foulds N, Cross NC, Aranaz P, Score J, Hidalgo-Curtis C, Robinson DO, Gibson J, Ennis S, Temple IK (2014). Megalencephaly syndromes: exome pipeline strategies for detecting low-level mosaic mutations. PLoS One.

[25] Barbieri CE, Baca SC, Lawrence MS, Demichelis F, Blattner M, Theurillat JP, White TA, Stojanov P, Van Allen E, Stransky N (2012). Exome sequencing identifies recurrent SPOP, FOXA1 and MED12 mutations in prostate cancer. Nat Genet.

[26] Bellacosa A, Kumar CC, Di Cristofano A, Testa JR (2005). Activation of AKT kinases in cancer: implications for therapeutic targeting. Adv Cancer Res.

[27] Manning BD, Cantley LC (2007). AKT/PKB signaling: navigating downstream. Cell.

[28] Phillips WA, Loconte DC, Grossi V, Bozzao C, Forte G, Bagnulo R, Stella A, Lastella P, Cutrone M, Benedicenti F (2015). Molecular and functional characterization of three different postzygotic mutations in PIK3CA-related overgrowth spectrum (PROS) patients: effects on PI3K/AKT/mTOR signaling and sensitivity to PIK3 inhibitors. PLoS One.

[29] Ehninger D, Han S, Shilyansky C, Zhou Y, Li W, Kwiatkowski DJ, Ramesh V, Silva AJ (2008). Reversal of learning deficits in a Tsc2+/− mouse model of tuberous sclerosis. Nat Med.

[30] Sato A, Kasai S, Kobayashi T, Takamatsu Y, Hino O, Ikeda K, Mizuguchi M (2012). Rapamycin reverses impaired social interaction in mouse models of tuberous sclerosis complex. Nat Commun.

